# Interaction between ENPP1 and homologous recombination deficiency defines distinct pan-cancer signatures: A retrospective observational study

**DOI:** 10.1097/MD.0000000000047164

**Published:** 2026-01-02

**Authors:** Yong Min Kim, Sung Hak Lee, Woong Na, Il Ju Lee, Mihye Kwon, Oyeon Jo, Young Soo Song

**Affiliations:** a Department of Pathology, College of Medicine, Konyang University, Daejeon, South Korea; b Department of Hospital Pathology, College of Medicine, The Catholic University of Korea, Seoul, South Korea; c Department of Pathology, H Plus Yangji Hospital, Seoul, South Korea; d LabGenomics Clinical Research Institute, LabGenomics, SeongNam, South Korea; e Department of Internal Medicine, College of Medicine, Konyang University, Daejeon, South Korea; f Myunggok Medical Research Center (Institute), College of Medicine, Konyang University, Daejeon, South Korea.

**Keywords:** ENPP1, functional enrichment analysis, HRD score, linear regression-based clustering, pan-cancer, survival analysis

## Abstract

Ectonucleotide pyrophosphatase/phosphodiesterase 1 (ENPP1), 1st identified in breast cancer and subsequently in multiple other cancer types, is an innate immune checkpoint regulator that recently emerged as a promising biomarker and therapeutic target. Homologous recombination deficiency (HRD) has gained clinical relevance with therapeutic vulnerability, particularly in breast and ovarian cancers. Despite the increasing significance of ENPP1 and HRD in cancer biology and treatment, their potential relationships have not yet been comprehensively investigated. We analyzed the relationship between ENPP1 expression and HRD score across the Cancer Genome Atlas pan-cancer and individual tumor types using the Pearson and Spearman correlations. To account for heterogeneity, pan-cancer samples were clustered using linear regression into 3 groups based on Bayesian Information Criterion. Differential expression, functional enrichment, and survival analyses were performed for these clusters at both the pan-cancer and representative tumor type levels. Although the pan-cancer relationship between ENPP1 expression and HRD score was heterogeneous, significant correlations were observed in 11 tumor types. Linear regression-based clustering resolved this heterogeneity into 3 functionally and clinically distinct groups: Cluster 1 was characterized by proliferation programs; Cluster 2 by extracellular matrix remodeling, differentiation, and immune response; and Cluster 3, by metabolic reprogramming. Clinically, Cluster 3 was associated with better survival than Clusters 1 and 2 in a pan-cancer analysis (*P* < .0001). At the individual tumor type level, these global cluster features were further modified in tissue-specific contexts, reflecting local microenvironment adaptation. Significant survival differences were observed in patients with adrenocortical carcinoma, chromophobe renal cell carcinoma, low grade glioma, and mesothelioma, further underscoring the tissue-specific modification of global cluster features. Our comprehensive pan-cancer analysis revealed the intrinsic heterogeneity of ENPP1 expression and HRD score, which may arise from complex and dynamic interactions with diverse cancer hallmarks, including proliferation, extracellular matrix remodeling, immune response, and metabolic reprogramming, and can be generalized into 3 clusters with distinct molecular and clinical characteristics. At the individual tumor type level, these global cluster features were further modified to adapt to a tissue-specific microenvironment, manifesting distinct tissue-specific patterns. Collectively, these findings provide a foundation for refining biomarker-driven precision medicine strategies for diverse tumor types.

## 1. Introduction

Ectonucleotide pyrophosphatase/phosphodiesterase 1 (ENPP1), an innate immune checkpoint that regulates the cyclic GMP–AMP, which is a stimulator of interferon genes pathway, has recently emerged as a promising biomarker in immuno-oncology with therapeutic potential through selective enhancement of anti-cancer immunity.^[1–11]^ Together with its therapeutic potential, the crucial features of ENPP1 were initially explored in breast cancer, and is now being actively investigated across multiple cancer types.^[9,12]^ Therefore, the interaction between ENPP1 and other key biomarkers is emerging as a major theme in cancer research.^[11]^

Homologous recombination is a complex DNA repair system comprising numerous components and tumors harboring homologous recombination deficiency (HRD) has emerged as important therapeutic target in various cancers particularly breast and ovarian cancers.^[13–23]^ The presence of HRD, mainly caused by germline or somatic mutations of HRD-related genes, is a crucial determinant in prescribing poly(ADP-ribose) polymerase inhibitors, which can induce synthetic lethality and ultimately improve patient survival.^[22,24,25]^ Although HRD can be assessed by various metrics, the HRD score is currently the most widely used in clinics.^[15,20,23]^

The potential interaction between ENPP1 expression and HRD score seems plausible, although, to our knowledge, a direct analysis of this relationship has not yet been reported. ENPP1 has been reported to be highly expressed in cancers characterized by genomic instability.^[26]^ Moreover when adenosing (ADO) signaling was assessed using an ADO score to which ENPP1 contributed significantly, a significant association was observed between the ADO score and presence of HRD.^[27]^ In contrast, HRD might attenuate ENPP1 activity by altering NAD+/ATP balance or by suppression of the cGAS-stimulator of interferon genes pathway.^[5,28]^ Furthermore tumors with HRD often exhibit immune-hot microenvironment in which ENPP1 activity might be lowered.^[29–31]^ In this context, to address the missing links between HRD and innate immune modulation, a comprehensive analysis of the relationships between ENPP1 expression and HRD score across multiple cancer types may be warranted.

In this study, using The Cancer Genome Atlas (TCGA) dataset,^[32]^ we investigated the relationship between ENPP1 expression and HRD scores across multiple cancer types and assessed their molecular and clinical significance. By applying linear regression-based clustering to pan-cancer data, the cases were naturally separated into 3 groups with distinct molecular and clinical features. These observations suggest that the interaction between innate immune regulation and DNA repair system is complex and widespread, and may hold promise as a novel biomarker for cancer stratification.

## 2. Methods

### 2.1. Data collection and preprocessing

This retrospective observational study adhered to the STROBE guidelines. For a comprehensive analysis of the relationship between ENPP1 gene expression and HRD score across 33 cancer types, TCGA data (normalized pan-cancer ribonucleic acid-Seq, HRD score, and clinical data) were retrieved and downloaded using the UCSC Xena browser.^[33]^ In accordance with TCGA polices, neither institutional ethics approval nor patient consent was necessary in this study. Because lists of TCGA sample IDs were not entirely consistent across data types, we initially created a pan-cancer dataset for ENPP1 expression and HRD score, and then survival data were assembled to this dataset. Consequently, 9828 samples were included in the analysis of the relationship between ENPP1 expression and HRD score, and survival analyses were performed on 9625 patients (Section 2.5). Molecular and survival analyses for individual cancer types were also performed using the same sample sets (see Table S1, Supplemental Digital Content, https://links.lww.com/MD/R134, which summarizes the number of samples per cancer type used in molecular and survival analyses).

### 2.2. Correlation between ENPP1 expression and HRD score

To evaluate the global pan-cancer relationship between ENPP1 expression and the HRD score, scatter plots were generated and the associations were quantified using both Pearson and Spearman correlation coefficients. The same analyses were independently performed for each tumor type. To enhance visualization of tumor type-specific territories within the pan-cancer scatter plot, we applied *k*-nearest neighbors approach (*k* = 50).^[34]^ By employing both correlation metrics (Pearson and Spearman correlation coefficients) in a complementary manner, we aimed to capture more reliable and robust correlation trends across the heterogeneous pan-cancer landscape as well as within individual cancer types.

### 2.3. Linear regression-based clustering

Linear regression-based clustering is a statistical technique that partitions an entire data into distinct clusters and then fits a separate linear regression model to each cluster.^[35,36]^ This approach offers greater flexibility when a single global linear regression model cannot adequately capture complex or heterogeneous data structures. Because the distribution of ENPP1 expression and HRD score did not exhibit clearly separable clusters or a single global linear relationship, linear regression-based clustering was considered an appropriate choice for this dataset. For implementation, we used the R package *flexmix* (version 2.3-20; Austria), which provides a general framework for fitting finite mixtures of regression models based on an expectation–maximization algorithm.^[37]^ The optimal number of clusters was determined using the Bayesian Information Criterion, as implemented in the same package.

To identify the unique characteristics of the clusters derived from linear regression-based clustering, 3 main analyses were performed. First, the information–theoretic framework was applied to quantitatively assess the dependence and independence relationships among the clusters, ENPP1 expression, and the HRD score. Second, the ENPP1 expression patterns for each cluster were analyzed in detail. Third, the results of this study’s clustering methods were compared with those obtained using *k*-means clustering, a commonly used unsupervised method.^[38]^

For the specific implementation of the information-theoretic framework, the *infotheo* R package was utilized to measure the entropy and mutual information of the clusters, ENPP1 expression, the HRD score, and their multivariate relationships.^[39]^ The presence of unique information in the clusters, independent of the individual effects of ENPP1 expression and the HRD score, was confirmed when the entropy of the clusters was greater than the mutual information between ENPP1 expression and the HRD score.^[40]^

### 2.4. Differentially expressed genes (DEGs) and gene set-based enrichment tests between clusters

To identify molecular features distinguishing the clusters derived from the linear regression-based clustering, DEGs were identified using the *limma* R package (version 3.60-6), applying selection criteria of absolute log2-fold change > 1 and adjusted *P*-value of < .05.^[41]^ The identified DEGs were subsequently analyzed using gene ontology (GO) enrichment and Kyoto Encyclopedia of Genes and Genomes (KEGG) pathway enrichment analyses implemented in the *ClusterProfiler* R package (version 4.16-0).^[42]^

These DEG analyses and molecular profiling using GO and KEGG were performed for both the pan-cancer cohort and individual cancer types. For the latter, instead of analyzing all 33 cancer types, we focused on the representative ones that exhibited significant survival differences according to the cluster membership (Section 2.5). Subsequent DEG and enrichment analyses were conducted for these selected cancer types to elucidate the biological mechanisms associated with their distinctive clinical outcomes.

### 2.5. Survival analysis

For the assessment of clinical relevance, survival analyses were performed using *survival* and *survminer* R packages.^[43,44]^ A total of 9828 tumor samples from TCGA were initially included in the primary analyses of ENPP1 expression and HRD score (Sections 2.2–2.4). Clinical survival data (overall survival time and vital status) were retrieved from TCGA Pan-Cancer Atlas clinical datasets. Samples were included in the survival analysis if both molecular data (ENPP1 expression and HRD score) and corresponding clinical survival information were available. Samples lacking either ENPP1 expression, HRD score, or survival data were excluded from further analysis.

After applying these inclusion and exclusion criteria, 9625 samples (patients) with complete molecular and survival information were retained, corresponding to 97.93% of the initial dataset. Because only 203 samples (2.07%) were excluded due to missing clinical data, the potential bias introduced by data incompleteness was considered minimal. This cohort of 9625 samples constituted the final dataset for all subsequent survival analyses.

A 2-tiered survival analysis was conducted across the pan-cancer cohort. First, in the pan-cancer cohort, Kaplan–Meier survival curves were compared among the established clusters derived from regression-based analysis. Statistical significance was assessed using the log-rank test. Second, Cox proportional hazards were applied to investigate the effect of cluster membership on survival. To control for potential confounding factors, tumor type or ENPP1 expression was included as covariates, allowing the evaluation of cluster-specific survival effects independent of tumor type and ENPP1 expression level.

To identify cancer types that were clinically aligned with the established pan-cancer-derived clusters, Kaplan–Meier survival analyses were performed for each of the 33 individual cancer types, following the same procedures applied to the pan-cancer dataset. Cancer type annotations were not available for 59 samples; therefore, a total of 9566 samples (patients) were included in the cancer type-specific survival analyses. Only cancer types exhibiting statistically significant survival differences among clusters were selected for subsequent molecular characterization using GO and KEGG pathway enrichment analyses (Section 2.4). Because sample sizes varied substantially across cancer types, the purpose of this selection was not to exclude cancer types lacking statistical significance in the current dataset, but rather to highlight those with distinctive survival patterns warranting further molecular investigation.

## 3. Results

### 3.1. Landscape of ENPP1 expression and HRD score

**Figure 1.**
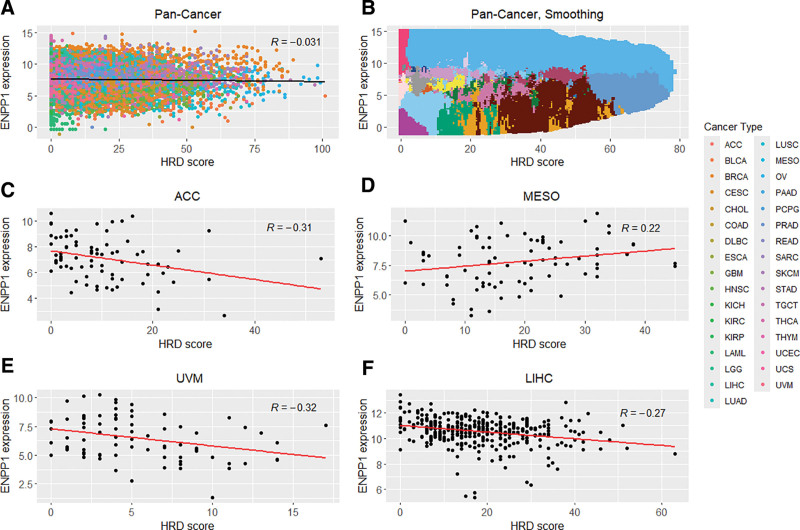
Distribution of ENPP1 expression and HRD score in pan-cancer and representative tumor types. (A) Scatterplot of ENPP1 expression versus HRD score across all TCGA tumor types, color-coded by tumor type. (B) Same as in (A), with tumor type territories clarified using *k*-nearest neighbors. (C–F) Tumor type-specific distributions in ACC (C), MESO (D), UVM (E), and LIHC (F). Black or red lines indicate linear regression fits. “R” denotes Pearson correlation coefficients. ACC = adrenocortical carcinoma, BLCA = bladder urothelial carcinoma, BRCA = breast invasive carcinoma, CESC = cervical squamous cell carcinoma and endocervical adenocarcinoma, CHOL = cholangiocarcinoma, COAD = colon adenocarcinoma, DLBC = lymphoid neoplasm diffuse large B-cell lymphoma, ENPP1 = ectonucleotide pyrophosphatase/phosphodiesterase 1, ESCA = esophageal carcinoma, GBM = glioblastoma multiforme, HNSC = head and neck squamous cell carcinoma, HRD = homologous recombination deficiency, KICH = kidney chromophobe renal cell carcinoma, KIRC = kidney renal clear cell carcinoma, KIRP = kidney renal papillary cell carcinoma, LAML = acute myeloid leukemia, LGG = brain low grade glioma, LIHC = liver hepatocellular carcinoma, LUAD = lung adenocarcinoma, LUSC = lung squamous cell carcinoma, MESO = mesothelioma, OV = ovarian serous cystadenocarcinoma, PAAD = pancreatic adenocarcinoma, PCPG = pheochromocytoma and paraganglioma, PRAD = prostate adenocarcinoma, READ = rectum adenocarcinoma, SARC = sarcoma, SKCM = skin cutaneous melanoma, STAD = stomach adenocarcinoma, TGCT = testicular germ cell tumors, THCA = thyroid carcinoma, THYM = thymoma, UCEC = uterine corpus endometrial carcinoma, UCS = uterine carcinosarcoma, UVM = uveal melanoma.

We explored the landscape of ENPP1 expression and HRD score using a scatter plot (Fig. 1). The Pearson and Spearman correlation coefficients of the 9828 pan-cancer samples were −0.03 and −0.06, respectively (Fig. 1A). When the cancer type-specific distribution of ENPP1 expression and HRD score were delineated using the *k*-nearest neighbor approach, each cancer type exhibited a unique territory (Fig. 1B). Nevertheless, mixed and heterogeneous regions were observed, particularly in low- to mid-level ENPP1 expression, and low HRD score range where prostate adenocarcinoma, colon adenocarcinoma, rectum adenocarcinoma, cervical squamous cell carcinoma, and kidney renal clear cell carcinoma were the predominant tumor types. Because the tumor type exerted a strong effect, correlations were also analyzed within each tumor type (see Table S2, Supplemental Digital Content, https://links.lww.com/MD/R134, which illustrates the results of correlation analysis between ENPP1 expression and HRD score for each tumor type). Eleven tumor types demonstrated statistically significant correlations in both the Pearson and Spearman correlation coefficients (Table 1 and Fig. 1C–F).

**Table 1:** Cancer types showing significant correlations between ENPP1 expression and HRD score.

Cancer type	Pearson correlation		Spearman correlation	
	Coefficient	*P*-value	Coefficient	*P*-value
LUAD	0.24	<.0001	0.23	<.0001
TGCT	0.22	.0055	0.20	.0107
PAAD	−0.20	.0120	−0.22	.0060
LIHC	−0.27	<.0001	−0.27	<.0001
CESC	−0.12	.0343	−0.12	.0360
BRCA	−0.28	<.0001	−0.22	<.0001
MESO	0.22	.0443	0.23	.0389
STAD	0.11	.0305	0.14	.0051
HNSC	0.11	.0107	0.10	.0234
ACC	−0.31	.0057	−0.33	.0033
UVM	−0.32	.0038	−0.33	.0027

ACC = adrenocortical carcinoma, BRCA = breast cancer, CESC = cervical squamous cell carcinoma, ENPP1 = ectonucleotide pyrophosphatase/phosphodiesterase 1, HNSC = head and neck squamous cell carcinoma, HRD = homologous recombination deficiency, LIHC = liver hepatocellular carcinoma, LUAD = lung adenocarcinoma, MESO = mesothelioma, PAAD = pancreatic adenocarcinoma, STAD = stomach adenocarcinoma, TGCT = testicular germ cell tumors, UVM = uveal melanoma.

### 3.2. Linnear regression-based clustering of HRD–ENPP1 data

**Figure 2.**
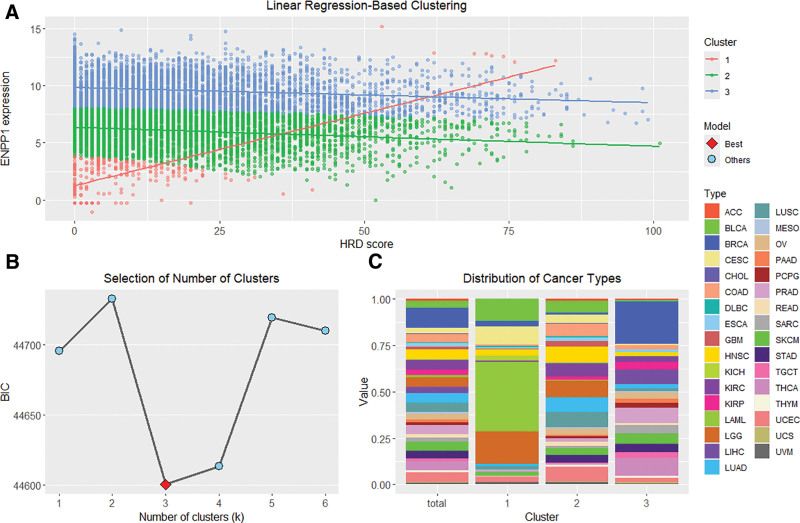
Linear regression-based clustering of ENPP1 expression and HRD score. (A) Scatter plot showing the linear regression-based clustering of pan-cancer samples into 3 distinct groups. Each cluster is represented by a different color, with regression lines fitted to ENPP1 expression versus HRD score within each cluster. (B) Model selection based on the Bayesian Information Criterion (BIC) identified 3 clusters as the optimal solution, as indicated by the lowest BIC value. (C) Bar plot showing the distribution of tumor types across the 3 clusters. Each cancer type is represented by a distinct color, illustrating heterogeneous contributions of tumor types to each cluster. ACC = adrenocortical carcinoma, BLCA = bladder urothelial carcinoma, BRCA = breast invasive carcinoma, CESC = cervical squamous cell carcinoma and endocervical adenocarcinoma, CHOL = cholangiocarcinoma, COAD = colon adenocarcinoma, DLBC = lymphoid neoplasm diffuse large B-cell lymphoma, ENPP1 = ectonucleotide pyrophosphatase/phosphodiesterase 1, ESCA = esophageal carcinoma, GBM = glioblastoma multiforme, HRD = homologous recombination deficiency, HNSC = head and neck squamous cell carcinoma, KICH = kidney chromophobe renal cell carcinoma, KIRC = kidney renal clear cell carcinoma, KIRP = kidney renal papillary cell carcinoma, LAML = acute myeloid leukemia, LGG = brain low grade glioma, LIHC = liver hepatocellular carcinoma, LUAD = lung adenocarcinoma, LUSC = lung squamous cell carcinoma, MESO = mesothelioma, OV = ovarian serous cystadenocarcinoma, PAAD = pancreatic adenocarcinoma, PCPG = pheochromocytoma and paraganglioma, PRAD = prostate adenocarcinoma, READ = rectum adenocarcinoma, SARC = sarcoma, SKCM = skin cutaneous melanoma, STAD = stomach adenocarcinoma, TGCT = testicular germ cell tumors, THCA = thyroid carcinoma, THYM = thymoma, UCEC = uterine corpus endometrial carcinoma, UCS = uterine carcinosarcoma, UVM = uveal melanoma.

As the relationships between ENPP1 expression and HRD score across pan-cancer samples appeared heterogeneous, we applied linear regression-based clustering to identify multiple plausible linear patterns between ENPP1 expression and HRD score (Fig. 2A). The number of clusters was set to 3 according to the Bayesian Information Criterion (Fig. 2B). Cluster 1 exhibited a positive linear relationship between ENPP1 expression and HRD score, whereas Clusters 2 and 3 showed slightly negative relationships. Generally, the samples in Cluster 3 showed higher ENPP1 expression than those in Cluster 2. The composition of tumor types was also heterogeneous among the 3 clusters (Fig. 2C).

We investigated the uniqueness of the cluster patterns from the individual influences of ENPP1 expression and HRD score, and assessed whether these clusters could be reproduced by conventional clustering methods using 3 distinct analytic approaches.

First, in the information-theoretic analysis of the pan-cancer data, the entropy values of the clusters, ENPP1 expression, and HRD score were 0.80, 3.04, and 2.94, respectively, whereas the pairwise mutual information between the clusters and ENPP1 expression, and between the clusters and HRD score were 0.66 and 0.02, respectively. These findings suggest that the cluster assignments capture synergistic information arising from the combined relationship between ENPP1 expression and HRD score, which is not accounted by single-marker linear associations.

Second, despite the fact that the separation between Clusters 2 and 3 was largely driven by ENPP1 expression (with a relatively modest contribution from the HRD score), substantial overlap in expression was observed. This overlap highlights the internal heterogeneity with the clusters. Specifically, approximately 37.0% (1907/5149) of Cluster 2 samples exhibited ENPP1 expression levels higher than the minimum expression of ENPP1 observed in Cluster 3. Conversely, approximately 10.7% (470/4408) of Cluster 3 samples showed ENPP1 expression lower than the maximum observed in Cluster 2. These quantitative findings indicate that, despite the overall higher ENPP1 expression in Cluster 3, the distinction between Clusters 2 and 3 cannot be made by ENPP1 expression alone. Additionally Cluster 1 was found to be entirely independent from the associations observed in Clusters 2 and 3.

Finally we compared our linear regression-based clustering results with those obtained using *k*-means clustering with the number of the clusters fixed at *k* = 3. The cluster assignments from *k*-means clustering showed substantial discordance with those from the regression-based clustering (see Table S3, Supplemental Digital Content, https://links.lww.com/MD/R134, which compares linear regression-based clustering results with those of *k*-means clustering).

Collectively, these findings confirm that the unique molecular and clinical characteristics captured by our linear regression-based clustering approach cannot be reproduced by single-marker analysis or conventional clustering methods. This highlights the distinctiveness and added value of our integrative strategy.

### 3.3. DEGs between the clusters

**Figure 3.**
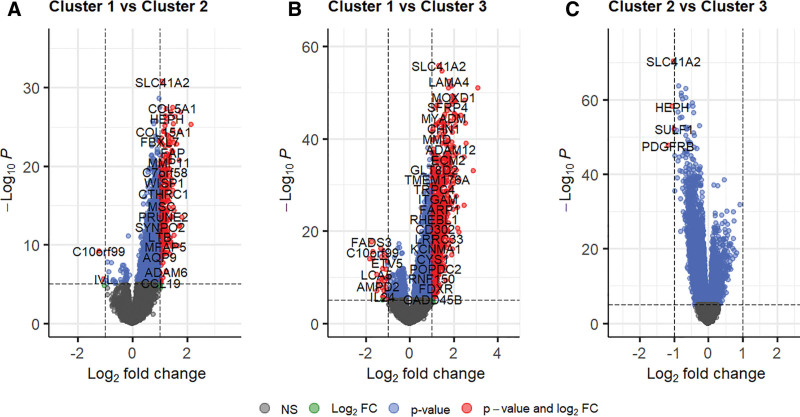
Volcano plots of pairwise comparisons of gene expression between the 3 clusters Gene expression comparisons are shown between (A) Clusters 1 and 2, (B) Clusters 1 and 3, and (C) Clusters 2 and 3. Vertical dotted lines indicate a log2-fold change of 1 or −1, while the horizontal dotted line indicates an adjusted *P*-value of .05. Each dot on the plot represents an individual gene, colored according to its significance (*P*-value) and expression change (log2-fold change). Gray dots represent non-significant genes (NS), blue dots represent genes with a significant *P*-value only, green dots represent genes with a significant log2-fold change only, and red dots represent genes significant for both *P*-value and log2-fold change.

To identify significant molecular features among the clusters, we performed DEG analysis using the limma R package (Fig. 3). Between Clusters 1 and 2, 253 DEGs were identified, including SLC41A2, COL5A1, and FNDC1 (Fig. 3A). Between Clusters 3 and 1, 859 DEGs were identified including SLC41A2, MOXD1, and AEBP1 (Fig. 3B). Five DEGs, SLC41A2, HEPH, PDGFRB, and ENPP1, were identified in Clusters 2 and 3 (Fig. 3C). Notably ENPP1 itself was included in all pairwise comparisons, suggesting that cluster membership was highly dependent on ENPP1 expression.

### 3.4. Functional profiling using GO and KEGG enrichment test

**Figure 4.**
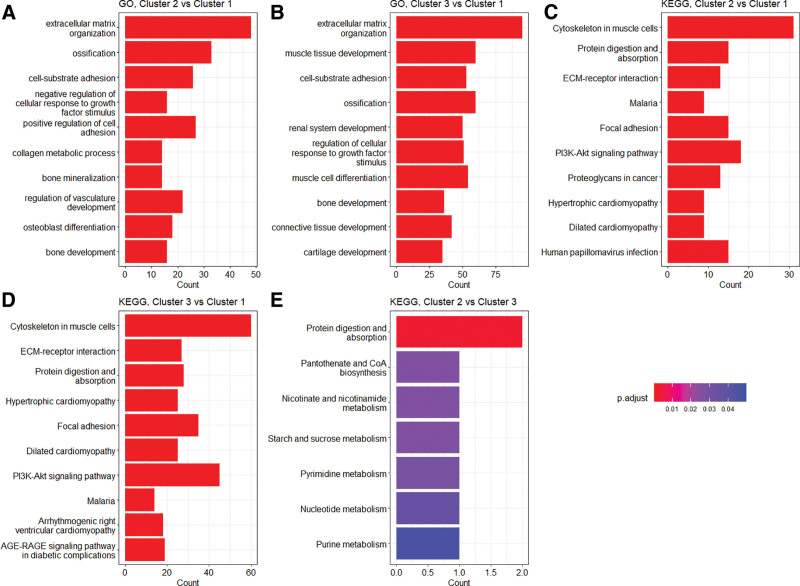
Functional enrichment analysis of differentially expressed genes (DEGs) between the clusters. This analysis was performed using Gene Ontology (GO) term enrichment or Kyoto Encyclopedia of Genes and Genomes (KEGG) pathway enrichment analysis. The 1st 2 bar plots represent the results of GO term enrichment analysis, comparing DEGs between (A) Clusters 1 and 2, and (B) between Clusters 1 and 3. The latter 3 bar plots represent the results of KEGG pathway enrichment analysis, comparing DEGs (C) between Clusters 1 and 2, (D) between Clusters 1 and 3, and (E) again between Clusters 2 and 3. If >20 terms were enriched, the top 20 enriched terms are displayed. The length of each bar indicates the count of DEGs belonging to the respective term or pathway, and the color represents the adjusted *P*-value (*P*.adjust). A deeper red color indicates a lower *P*-value, signifying higher statistical significance.

We investigated the functional characteristics of the DEGs between the clusters using GO term and KEGG pathway analyses. In the GO biologic process category, enriched terms between Clusters 1 and 2 included extracellular matrix (ECM) organization, extracellular structure organization, and regulation of leukocyte migration (Fig. 4A; see Table S4, Supplemental Digital Content, https://links.lww.com/MD/R134, which illustrates the results of GO term enrichment analysis between Clusters 1 and 2). Between Clusters 1 and 3, the enriched terms included ECM organization, extracellular structure organization, and renal tissue development (Fig. 4B; see Table S5, Supplemental Digital Content, https://links.lww.com/MD/R134, which illustrates the results of GO term enrichment analysis between Clusters 1 and 3). Between Clusters 2 and 3, the enriched terms included symbiont adhesion to a host cell, negative regulation of glycogen biosynthetic process, and ECM organization (see Table S6, Supplemental Digital Content, https://links.lww.com/MD/R134, which illustrate the results of GO term enrichment analysis between Clusters 2 and 3). Collectively, the GO enrichment analysis result suggested that Clusters 2 and 3 were functionally more similar than to Cluster 1.

In the KEGG pathway enrichment analysis, enriched pathways between Clusters 1 and 2 included ECM–receptor interaction, proteoglycans in cancer, and viral protein interaction with cytokine and cytokine receptor (Fig. 4C; see Table S7, Supplemental Digital Content, https://links.lww.com/MD/R134 which illustrates the results of KEGG pathway enrichment analysis between Clusters 1 and 2). Between Clusters 1 and 3, enriched pathways included ECM–receptor interaction, proteoglycans in cancer, and calcium signaling pathway (Fig. 4D; see Table S8, Supplemental Digital Content, https://links.lww.com/MD/R134 which illustrates the results of KEGG pathway enrichment analysis between Clusters 1 and 3). Between Clusters 2 and 3, enriched pathways included protein digestion and absorption, starch and sucrose metabolism, and nicotinate and nicotinamide metabolism (Fig. 4E; see Table S9, Supplemental Digital Content, https://links.lww.com/MD/R134 which illustrates the results of KEGG pathway enrichment analysis between Clusters 2 and 3). Consistent with the GO enrichment analysis, the KEGG pathway analysis suggested that Clusters 2 and 3 were functionally more similar to each other than to Cluster 1. However, they diverged in their dominant programs: Cluster 3 was enriched for multiple metabolic processes including glycogen and nucleotide metabolism, whereas Cluster 2 showed enrichment in the ECM and stromal organization, as well as host–symbiont interaction-related terms, which may reflect microenvironmental and defense-related biology.

### 3.5. Survival analysis

**Figure 5.**
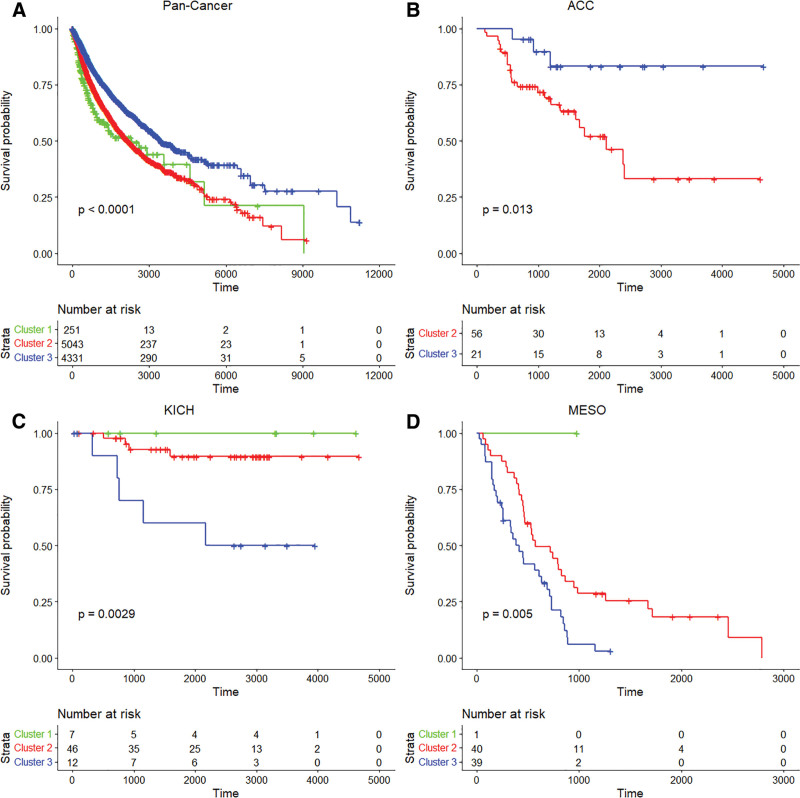
Survival analysis (overall survival) on the effect of cluster membership in pan-cancer or individual tumor types. Kaplan–Meier curves are displayed for patients with (A) pan-cancer, (B) adrenocortical carcinoma (ACC), (C) kidney chromophobe (KICH), and (D) mesothelioma (MESO).

We assessed the clinical significance of the 3 clusters, which reflects the interaction between ENPP1 expression and HRD score, by performing survival analysis using the Kaplan–Meier curves and Cox regression models (Fig. 5A). In the pan-cancer analysis, patients in Cluster 3 demonstrated significantly better survival than those in Clusters 1 and 2 (*P* < .0001). In the Cox regression models, cluster membership remained partially significant after adjusting for tumor type. Using Cluster 2 as a reference, Cluster 1 was associated with improved survival (HR = 0.63, *P* = .004), whereas Cluster 3 was no longer statistically significant (HR = 1.09, *P* = .07). Since ENPP1 expression has been reported to be a predictive factor in many tumor types, we included it as a covariate. Cluster membership remained significant after adjusting for ENPP1 expression. Specifically, compared with Cluster 2, Cluster 1 was associated with a reduced mortality risk (HR = 0.74, *P* = .027), and Cluster 3 also showed significantly lower risk (HR = 0.87, *P* = .007).

We further investigated the tumor types that showed significant differences in survival according to cluster membership. Four tumor types: adrenocortical carcinoma (ACC; Fig. 5B), kidney chromophobe renal cell carcinoma (KICH; Fig. 5C), low grade glioma (LGG; see Figure S1, Supplemental Digital Content, https://links.lww.com/MD/R135, which illustrates survival analysis between clusters in LGG), and mesothelioma (MESO; Fig. 5D), exhibited significant differences in Kaplan–Meier curve analyses (*P* = .013, *P* = .0029, *P* < .0001, and *P* = .005, respectively).

### 3.6. Functional profiling of individual tumor types

**Figure 6.**
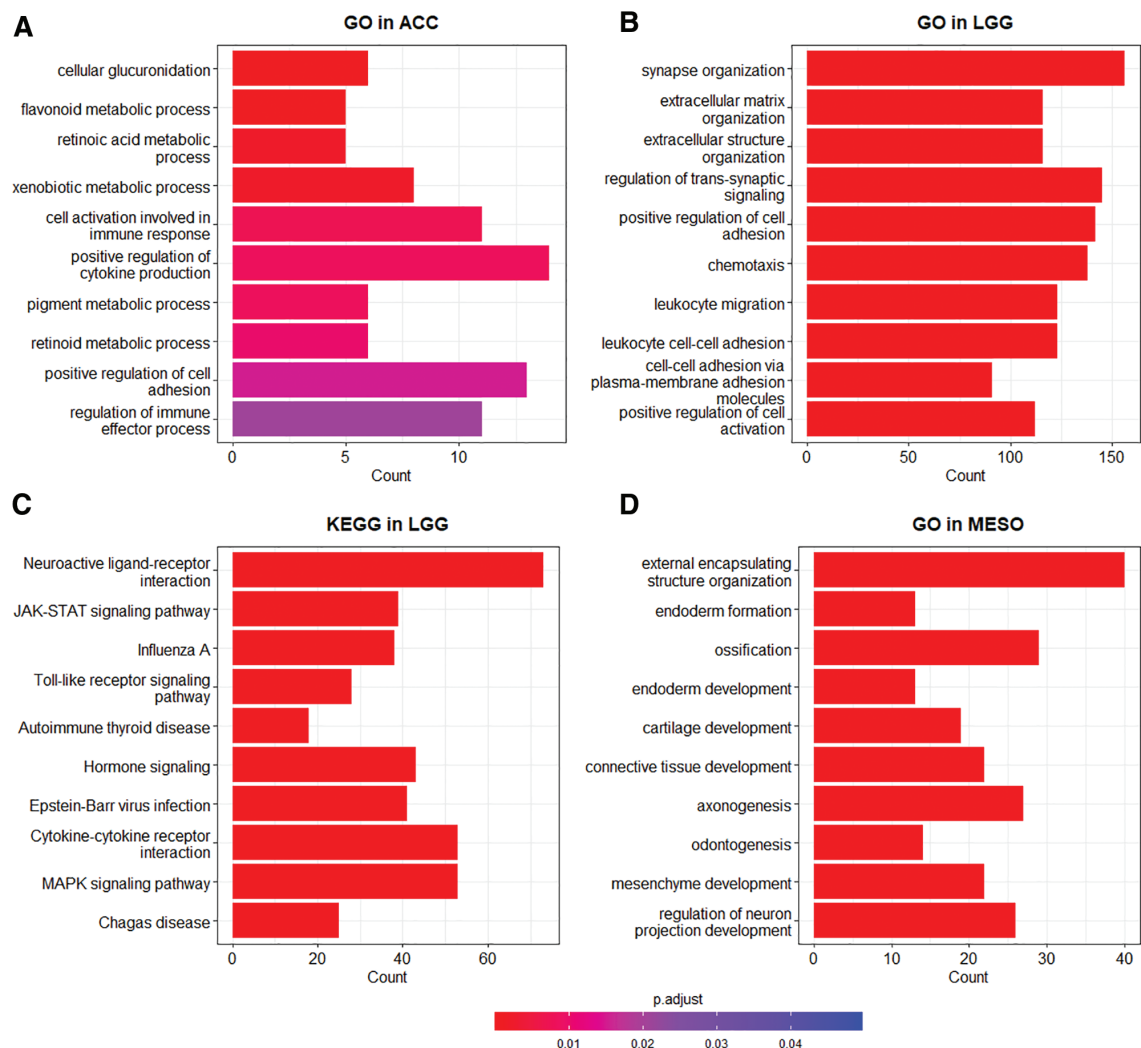
Functional enrichment analysis of differentially expressed genes (DEGs) between Clusters 2 and 3 in individual tumor types. This analysis was performed using the same method as in Figure 4. But applied to individual tumor types instead of pan-cancer samples. The 1st bar plot shows the results of Gene Ontology (GO) enrichment analysis (A) in adrenocortical carcinoma (ACC). The 2nd and 3rd bar plots display the results of (B) GO and (C) Kyoto Encyclopedia of Genes and Genomes (KEGG) pathway enrichment analyses in low grade glioma (LGG). The 4th bar plot presents the results of (D) GO enrichment analysis in mesothelioma (MESO). If >20 terms were enriched, the top 20 enriched terms are displayed. The length of each bar indicates the count of DEGs belonging to the respective term or pathway, and the color represents the adjusted *P*-value (*P*.adjust). A deeper red color indicates a lower *P*-value, signifying higher statistical significance.

To explore tumor type-specific molecular features associated with cluster membership, we performed GO term enrichment and KEGG pathway analyses for tumor types that showed significant survival differences (ACC, KICH, LGG, and MESO) using the same approach as in the pan-cancer analysis (Fig. 6). Because of the extremely small number of Cluster 1 samples in these tumor types, comparisons were restricted to a comparison between Clusters 2 and 3. Functional enrichment analysis revealed distinct tumor type-specific patterns and shared processes. ACC was characterized by enrichment of pathways related to metabolic processes, immune responses, and hormonal responses (Fig. 6A; see Table S10, Supplemental Digital Content, https://links.lww.com/MD/R134, which illustrates the results of GO term enrichment analysis between Clusters 2 and 3 in ACC, and Table S11, Supplemental Digital Content, https://links.lww.com/MD/R134, which illustrates the results of KEGG pathway enrichment analysis between Clusters 2 and 3 in ACC). In KICH, enriched pathways were predominantly associated with carbohydrate and nucleotide metabolism, insulin signaling, and energy homeostasis (see Table S12, Supplemental Digital Content, https://links.lww.com/MD/R134, which illustrates the results of GO term enrichment analysis between Clusters 2 and 3 in KICH, and Table S13, Supplemental Digital Content, https://links.lww.com/MD/R134, which illustrates the results of KEGG pathway enrichment analysis between Clusters 2 and 3 in KICH). LGG was enriched for immune responses, ECM regulation, and neuron/synapse programs (Fig. 6B and C; see Table S14, Supplemental Digital Content, https://links.lww.com/MD/R134, which illustrates the results of GO term enrichment analysis between Clusters 2 and 3 in LGG, and Table S15, Supplemental Digital Content, https://links.lww.com/MD/R134, which illustrates the results of KEGG pathway enrichment analysis between Clusters 2 and 3 in LGG). MESO demonstrated enrichment in mesenchyme development, ECM organization and response, and the transforming growth factor-beta signaling pathway (Fig. 6D; see Table S16, Supplemental Digital Content, https://links.lww.com/MD/R134, which illustrates the results of GO term enrichment analysis between Clusters 2 and 3 in MESO, and Table S17, Supplemental Digital Content, https://links.lww.com/MD/R134, which illustrates the results of KEGG pathway enrichment analysis between Clusters 2 and 3 in MESO).

## 4. Discussion

In this study, we comprehensively investigated the relationship between ENPP1 expression and HRD scores, and their molecular and clinical significance in pan-cancer samples. To overcome the intrinsic heterogeneity of ENPP1 expression and HRD score, we applied a linear regression-based clustering approach that delineated 3 distinct and biologically plausible clusters. Molecular functional profiling revealed that these clusters, identified from the pan-cancer samples, exhibited both cancer type nonspecific global characteristics and tumor type-specific features, thereby providing a framework for understanding how ENPP1 and HRD jointly shape tumor biology. This integrative analysis highlights the potential avenues for refined precision medicine strategies beyond single-gene or single-feature assessments.

In the pan-cancer samples, Cluster 1 was characterized by tumor cell-intrinsic proliferation programs, reflected by enrichment of GO terms or the KEGG pathways closely associated with the cell cycle, DNA replication, ribonucleic acid processing, oxidative phosphorylation, and mitochondrial translation. Cluster 2 exhibited signatures of ECM remodeling, development and differentiation programs, and immune responses. Finally, Cluster 3 was distinguished by tumor metabolic reprogramming, specifically enriched in glycogen biosynthetic processes, glucose import regulation, phosphate ion homeostasis, nucleoside triphosphate catabolic process, and triglyceride storage.

Although Cluster 1 could not be sufficiently characterized at the individual tumor type level owing to the extremely small number of samples, global molecular features of Clusters 2 and 3 were partly recapitulated in tumor type-specific analyses. In ACC, Clusters 2 and 3 diverged in pathways related to metabolic processes, immune responses, and hormonal regulation. While the enrichment of metabolic processes in Cluster 3 and immune response in Cluster 2 was largely consistent with the pan-cancer characteristics of these clusters, additional enrichment of hormonal regulation appeared to be tumor type-specific, suggesting that adrenal-specific metabolic and endocrine programs may uniquely manifest in ACC. Indeed, Wang et al recently reported that ACC exhibits pronounced metabolic heterogeneity, particularly in pathways such as the pentose phosphate and galactose metabolism, and that higher intratumoral metabolic variability correlated with poor survival outcomes.^[45]^ Moreover, cortisol-secreting ACC has been shown to exert systemic immunosuppression, further linking endocrine activity to immune escape and progrnosis.^[46–48]^ These findings further suggest that the observed cluster distinctions derived from ENPP1 expression and HRD score capture adrenal-specific tumor biology and may serve as clinically relevant biomarkers.

In KICH, Cluster 2 was preferentially enriched in immune-related pathways, whereas Cluster 3 was associated with metabolic processes, reflecting the global pan-cancer features of these clusters. These results suggest that tumor type-specific features between the clusters may further emerge once a sufficient number of Cluster 1 samples become available in KICH, and more comprehensive pairwise comparisons can be made. Despite the lack of Cluster 1 samples, the distinct features of Clusters 2 and 3 in KICH appear robust. A recent integrative study identified cell-cycle regulator CENPE and glycolytic enzyme LDHA as prognostic biomarkers in KICH, with LDHA expression strongly linked to metabolic rewiring and worse overall survival (HR = 1.10, *P* < .0001).^[49]^ These findings are consistent with our observation that metabolic dominance in Cluster 3 is associated with a poorer prognosis relative to the immune/ECM-enriched Cluster 2.

In the LGG, Cluster 2 was enriched in immune and inflammatory signaling, whereas Cluster 3 was enriched in neuronal and synaptic processes. These divergences suggest that the global pan-cancer immune/ECM-metabolic pattern is modified into a more brain tissue-specific representation of the cluster features, reflecting the unique neural tumor-microenvironment context of LGG. Previous studies have demonstrated that neuronal signaling pathways can contribute to glioma progression through neuron-glioma synaptic interactions.^[50]^ Thus, the balance between immune suppression and neural activity may underlie the distinct survival differences between clusters in LGG.

Finally, in MESO, Cluster 2 was enriched for immune activation, whereas Cluster 3 displayed signatures linked to cell adhesion and ECM remodeling. These results provide another example of the modification of global to tumor type-specific cluster features, reflecting the stromal-rich biology of mesothelioma, where ECM and transforming growth factor-beta pathway activation have been consistently implicated in tumor aggressiveness and immune exclusion.^[51–53]^

Although.Cluster 3 was associated with favorable survival outcomes in the pan-cancer analysis, tumor type-specific analyses (ACC, KICH, LGG, and MESO) revealed divergent patterns. For example, in ACC, patients in Cluster 3 showed superior survival compared with those in Cluster 2 (*P* = .013), whereas the opposite trend were observed in KICH (*P* = .003), LGG (*P* < .0001), and MESO (*P* = .005). This discrepancy may reflect tissue-specific modification of global clusters features, in which even subtle change may be sufficient to reverse survival outcomes. Furthermore, the heterogeneous distribution of cluster membership across tumor types may have contributed to these divergent survival patterns. These findings underscore that the biological and clinical implications of ENPP1-HRD-defined clusters are highly context-dependent and shaped by both global cancer hallmarks and tissue-specific programs.

This study has several limitations. First, linear regression-based clustering, which we used as the core strategy to resolve the heterogeneity of ENPP1 expression and HRD score, does not guarantee fully consistent cluster assignments because of the stochastic nature of the algorithm. Consequently, the exact cluster membership of the individual samples may not be reproducible. Nevertheless, despite such stochastic variations, the overall clustering patterns were predominantly preserved, and only a small fraction of the samples showed inconsistent membership across iterations. Further methodological advances are required to develop clustering algorithms that provide more stable and reproducible cluster construction. Second, owing to the inherent heterogeneity across tumor types and wide variation in sample size among them, a small subset of tumor types with relatively large cohorts and strong influence may have disproportionately driven the clustering patterns. This imbalance could reduce the generalizability of our findings across all tumor types, limiting the degree to which the clusters represent pan-cancer features rather than being dominated by high-impact tumor types. These issues may be partly mitigated if our approach is applied to groups of tumors that are more homogeneous in terms of sample size and underlying tumor biology. This refinement is a valuable direction for future studies.

Further investigation is required to translate our conceptual framework into clinically actional predictive or therapeutic biomarkers. These efforts include multi-omics characterization, evaluation of temporo-spatial tumor dynamics, and validation in clinically compatible materials such as formalin-fixed paraffin-embedded tissues. If ENPP1 expression can be reliably quantified as RT-qPCR ΔCt values from formalin-fixed paraffin-embedded samples and integrated with the established HRD score (already implemented clinically in breast and ovarian cancers) our 3-cluster framework could be feasibly adapted for precision oncology applications. The relatively low levels of intratumoral spatial heterogeneity observed for HRD scores further supports their potential clinical applicability.^[54]^

The divergence of the 3 clusters (representing proliferation, ECM-immune, metabolic reprogramming phenotypes) also suggests differential cluster-specific responses for cytotoxic agents, immunotherapies,^[12]^ and metabolism-targeted drugs.^[55]^ Identification of reliable biomarkers reflecting these cluster-specific biological programs could enable rational design of clinical trials tailored to each subgroups. Importantly, predictive or therapeutic biomarkers are not expected to be limited to ENPP1 or HRD themselves, but may include additional molecules that represent the broader molecular network underlying the 3-cluster framework.

In conclusion, we comprehensively analyzed the relationship between ENPP1 expression and HRD score across pan-cancer samples from 33 tumor types, which, to our knowledge, represents the first report on a pan-cancer scale. Furthermore, we addressed the intrinsic heterogeneity of this relationship by applying a linear regression-based clustering approach, which delineated 3 biologically and clinically distinct clusters. Functional enrichment analyses revealed global pan-cancer characteristics and tumor type-specific modifications in these clusters, highlighting the context-dependent interplay between DNA repair and innate immune regulation. The interaction between ENPP1 expression and HRD score appeared dynamic, context-dependent, and integrated into core cancer hallmarks rather than being fixed or isolated. Importantly, cluster membership was associated with distinct survival outcomes in both pan-cancer and individual tumor type analyses, underscoring their potential prognostic and biological relevance. Taken together, our findings provide novel insights into the ENPP1–HRD axis and suggest that these clusters may serve as a foundation for refining biomarker-driven precision medicine strategies across diverse tumor types.

## Author contributions

**Conceptualization:** Young Soo Song, Yong Min Kim, Sung Hak Lee, Woong Na, Oyeon Jo.

**Data curation:** Yong Min Kim, Sung Hak Lee, Oyeon Jo.

**Formal analysis:** Young Soo Song, Sung Hak Lee, Woong Na, Il Ju Lee.

**Funding acquisition:** Young Soo Song.

**Investigation:** Mihye Kwon, Oyeon Jo.

**Methodology:** Young Soo Song, Yong Min Kim, Sung Hak Lee, Woong Na, Il Ju Lee.

**Project administration:** Yong Min Kim, Il Ju Lee, Oyeon Jo.

**Resources:** Sung Hak Lee.

**Supervision:** Young Soo Song, Sung Hak Lee, Mihye Kwon.

**Validation:** Young Soo Song, Yong Min Kim, Sung Hak Lee, Oyeon Jo.

**Visualization:** Young Soo Song, Yong Min Kim, Il Ju Lee, Mihye Kwon.

**Writing – original draft:** Young Soo Song, Yong Min Kim, Sung Hak Lee, Woong Na, Il Ju Lee, Mihye Kwon, Oyeon Jo.

**Writing – review & editing:** Young Soo Song, Yong Min Kim, Sung Hak Lee, Woong Na, Il Ju Lee, Mihye Kwon, Oyeon Jo.

## Supplementary Material




