# Supplementary material for: Interaction between ENPP1 and homologous recombination deficiency defines distinct pan-cancer signatures: A retrospective observational study
Source: Medicine (Baltimore). 2026 Jan 2;105(1):e47164. doi: 10.1097/MD.0000000000047164 (PMC12778157; doi:10.1097/MD.0000000000047164)

**Supplementary Figure S1. Survival analysis (overall survival) between clusters in low grade glioma (LGG). A Kaplan–Meier curve is displayed for patients with LGG.**


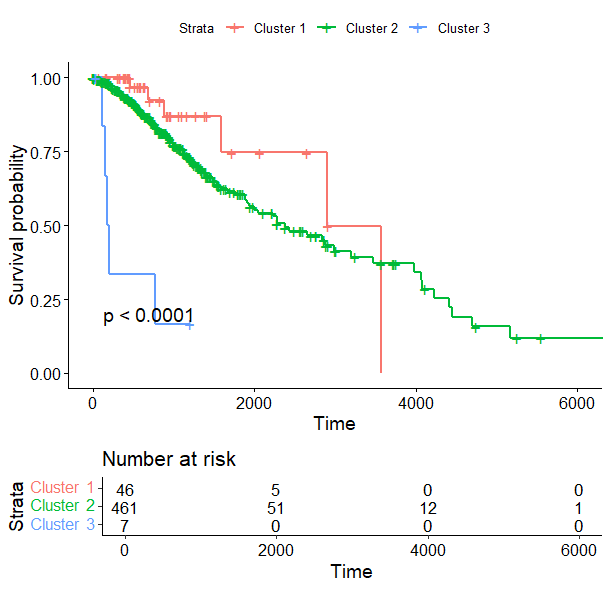

Supplement: Supplementary file 2 [file medi-105-e47164-s002.docx]
